# Universal Markers Unveil Metastatic Cancerous Cross-Sections at Nanoscale

**DOI:** 10.3390/cancers14153728

**Published:** 2022-07-31

**Authors:** Evangelos Bakalis, Angelo Ferraro, Vassilios Gavriil, Francesco Pepe, Zoe Kollia, Alkiviadis-Constantinos Cefalas, Umberto Malapelle, Evangelia Sarantopoulou, Giancarlo Troncone, Francesco Zerbetto

**Affiliations:** 1 Dipartimento di Chimica “G. Ciamician”, Università di Bologna, 2 Via Francesco Selmi, 40126 Bologna, Italy; francesco.zerbetto@unibo.it; 2 Theoretical and Physical Chemistry Institute, National Hellenic Research Foundation, 48 Vassileos Constantinou Avenue, 11635 Athens, Greece; an.ferraro@libero.it (A.F.); vgavriil@eie.gr (V.G.); zkollia@eie.gr (Z.K.); ccefalas@eie.gr (A.-C.C.); esarant@eie.gr (E.S.); 3 Dipartimento di Sanità Pubblica, Università degli Studi di Napoli “Federico II”, 5 Via Pansini, 801301 Napoli, Italy; francesco.pepe4@unina.it (F.P.); umberto.malapelle@unina.it (U.M.); giancarlo.troncone@unina.it (G.T.)

**Keywords:** atomic force microscopy imaging, metastatic cancer, multifractal analysis

## Abstract

**Simple Summary:**

We propose the use of two universal morphometric indices whose synergetic potency leads to the classification of a cancerous tissue of a few nanometers in size as metastatic or non-metastatic. The method is label-free, operates on conventional histological cross-sections, recording surface height–height roughness by AFM, and detects nanoscale changes associated with the progress of carcinogenesis which are the output of combined statistical approaches, namely multifractal analysis and the generalized moments method. The benefit of this approach is at least two-fold. On the one hand, its application in the context of early diagnosis can increase the life expectancy of patients, and on the other hand, differentiation between metastatic and non-metastatic tissues at the singular cell level can lead to new methodologies to treat cancer biology and therapies.

**Abstract:**

The characterization of cancer histological sections as metastatic, M, or not-metastatic, NM, at the cellular size level is important for early diagnosis and treatment. We present timely warning markers of metastasis, not identified by existing protocols and used methods. Digitized atomic force microscopy images of human histological cross-sections of M and NM colorectal cancer cells were analyzed by multifractal detrended fluctuation analysis and the generalized moments method analysis. Findings emphasize the multifractal character of all samples and accentuate room for the differentiation of M from NM cross-sections. Two universal markers emphatically achieve this goal performing very well: (a) the ratio of the singularity parameters (left/right), which are defined relative to weak/strong fluctuations in the multifractal spectrum, is always greater than 0.8 for NM tissues; and (b) the index of multifractality, used to classify universal multifractals, points to log-normal distribution for NM and to log-Cauchy for M tissues. An immediate large-scale screening of cancerous sections is doable based on these findings.

## 1. Introduction

Metastasis is the spread of cancer cells from the primary point of appearance to surrounding tissues and to distant organs. It is a complex process and involves alterations in the shape of cancer cells as well in their attachment to surrounding normal cells and the extracellular matrix (ECM) [1,2,3,4,5,6]. It is responsible for approximately 90% of cancer deaths, and its early prediction is fundamental for the patient survival rate, as well for the understanding of cancer biology [7,8]. Moreover, early prediction contributes to reducing the metastatic expansion of cancerous cells through targeting strategies [9]. Tissues’ morphological factors and biomechanical properties have been used to identify metastatic cancers, and to some extent, they achieve their goal [9,10]. Histological, cytological, and biomarker-based analysis provide an assessment of the cancer stage, as well of the clinical variables which are used for prognostic insights, whose stratification risk value, however, is insufficient for predicting survival outcomes and metastatic prognostic value [11].

Cancer tissues are characterized by irregular growth and the features of their geometrical changes are far from being attributed by Euclidean measures (length, surface, volume), which deliver gross differences for geometrical objects and losing details of their structure. Instead, fractal geometry is able to decode fine differences among structures. Fractals are ubiquitous in nature, and are consequences of self-affinity resulting in scale-independent processes [12,13,14]. Fractal analysis is already used to study cancer [15,16], and it can lead to the improvement of the diagnostic validity of both cyto-histopathological and medical imaging findings [17]. For instance, fractal analysis has been used to elucidate various types of tumors: colorectal cancer (CRC) [18]; rectal cancer [19]; and breast cancer [20,21,22], to name a few. The fractal or Hausdorff dimension (FD) [23] is the index that has been used the most in cancer analysis. In addition to FD, the texture properties (lacunarity) of tissues have also been investigated. FD and lacunarity assess the degree of complexity in patterns [24], are based on structural (shape, distribution of gaps) complexity and statistical properties of geometrical objects. FD and lacunarity are good descriptors in discriminating cancerous tissues from normal ones; for instance, the multifractal analysis of digitized mammograms showed monofractal behavior for normal tissues and the multifractal character of clustered microcalcifications, and breast cancer imprints [25,26]. Additionally, the FD of atomic force microscopy (AFM) images can differentiate premalignant from malignant human cervical epithelial cells [27].

Various mathematical models deliver the FD [28], and can potentially lead to a range of estimates for its value [29]. FD quantifies the roughness or smoothness of time series and spatial data in the limit of infinitesimally fine observation scale, and thus the estimates of FD depend on the availability of observations at sufficient fine temporal or spatial resolution [30]. However, for a broad range of potentially anisotropic, non-stationary, as well some non-Gaussian processes, FD can be considered as second-order statistics [31,32]. Second-order statistics—in the same class as which measures such as lacunarity, auto/cross-correlation, and power spectrum fall—well describe monofractal processes and their ability to discriminate tiny differences between multifractal processes are questionable since their estimates are rough because of describing a multifractal around its mean value [33].

Fractal analysis showed that cancer tissues are better described by multifractals [18,24,34]. However, cancer cells are heterogeneous both phenotypic and functional within the same tumor as a consequence of either genetic changes, or reversible changes in the ECM, or environmental differences [35]. It is thus expected metastatic (M) and non-metastatic (NM) tissues will exhibit multifractal behavior likely accompanied by anisotropy in scaling (multiaffinity) along the Cartesian axes used to describe the sample tissue. Multiaffine surfaces are better described by markers such as the multifractal spectrum, the singularity strength, the generalized Hurst exponent, whose ability of description is not limited to a single value but they rather provide a spectrum of values [36]. For example, longitudinal whole-body PET-CT scans of F-18-fluorodeoxyglucose in patients suffering from metastatic melanoma have been analyzed by multifractal analysis and its multifractal spectrum delivers info on the distribution of the test molecule around metastatic centers and its value decreases with disease progression [17].

A reliable and label-free approach to identify and quantify M and NM tissues on conventional histological cross-sections, and thus to detect nanoscale changes associated with the progress of carcinogenesis in tissue for early diagnosis and effective treatment is challenging [37]. To reduce the size of the histological cross-sections to be analyzed, AFM is a powerful technique because of performing direct three-dimensional imaging of cells and tissues going far beyond the resolution limits of optical microscopes. The main abilities of AFM, force spectroscopy (nanoindentation) and topography imaging, have been exploited in cancer research. For example, the connection between the motility and mechanics of human glioblastoma (GBM) cells was analyzed using AFM imaging in a live cell [38], and single cell live imaging was also used to quantify biomechanical and migratory properties in low- and high-metastasis-associated in colon cancer 1 marker (MACC1)-expressing CRC cells [39]. AFM force experiments have also been carried out to estimate cell stiffness in MDA-MB-231 breast cancer cells [40], as well to investigate the combined influence of a glioma isocitrate dehydrogenase (IDH) mutation status on both tumor and peritumoral white matter fresh tissue elasticity [41]. The use of AFM in cancer research potentially reveals structural changes in tissues, which can be associated with the early cancer progression at the nanoscale [42], as can also be seen in two recent reviews [43,44].

Up to now, few studies have focused on the analysis of formalin-fixed paraffin-embedded (FFPE) cancer tissues using AFM [45], and in particular, to use the extracted information to improve diagnosis and prognosis reports [46]. In the present work, M and NM histological cross-sections of CRC tissues were prepared, and their assessment (FFPE sections) was performed by optical microscope. The morphological features of a small part of them, with a surface size up to few cancer cells, were scanned by AFM operating at tapping mode, and the histological cross-section height–height roughness was recorded. Multifractal detrended fluctuation analysis (MF-DFA) and fluctuation analysis by means of the generalized moments method (GMM) in two dimensions were used. We found that the differences between M and NM primary tissues are minute; however, our combined methodology can discriminate M from NM tissues based on the information extracted by processing AFM images. Indeed, our results shown that it is possible to classify tumors as M or NM by just analyzing the primary tumor tissues, in this case, colon sections, without any further information about regional lymph nodes or the analysis of other organs. Our method provides insights into tumors’ metastatic potential by answering the malignancy of suspicious cells (typically a few) not identified with the optical examination when signs of early disease are subtle.

## 2. Materials and Methods

### 2.1. Histological Tissue Preparation

CRC tissues were obtained from the University Hospital “Federico II” in Naples, Italy. All information regarding human material was managed using anonymous numerical codes and all samples were handled in compliance with the Declaration of Helsinki protocol and approved by the Institutional Review Board (or Ethics Committee) of Universita’ Federico II, Napoli, Italy, on 13 June 2018. For each patient, the site of the primary tumor (right colon, transverse, left colon, rectosigmoid), the pathological classification according to the Union for International Cancer Control (UICC) 2017 (T, N, M), the presence of vascular hematic invasion (V), vascular, lymphatic invasion (L), and perineural invasion (Pn), and surgical resection margins (R) status were reported. Moreover, the neoplastic cellular percentage, the presence of necrosis, desmoplasia, and tumor-infiltrating lymphocytes (TILs) was evaluated by microscopic visual inspection by dedicated pathologists. The acellular mucinous component was categorized as absent (<1%) or present (≤50% or >50%) after the microscopic revision performed by two expert gastrointestinal pathologists. The TNM classifications of tumor samples used in the study is as follows; m1: pT3NXM1; m2: pT4ApN1ApM1; m3: pT3N2bM1b; nm1: pT4aN0; nm2 pT3N0.

FFPE blocks have been prepared by the following routine protocol. Freshly dissected tissues were fixed with 10% neutral formalin for 24 hours at room temperature. Tissues were rinsed with running tap water for 1 hour, dehydrated through 70%, 80%, and 95% alcohol, 10 min each, followed by three changes of 100% alcohol, 10 min each. Subsequently, tissues were cleared through two changes of xylene, 10 min each; and two changes in liquid paraffin, 10 min each. Finally, the tissues were embedded in a paraffin block. All blocks were stored at room temperature in the hospital pathological tissue archive. The tumor sections in FFPE blocks, collected on glass slides, were dewaxed according to the following procedure: warmed at 60 °C until paraffin completely melted down; then, three series of xylene washes were performed, leaving 5 min the glass slides completely submerged and changing xylene at each step; then, xylene traces were removed by three series of wash in 100% ethanol for 5 min each time and changing ethanol solution at each step; then, slides were further washed once in 95% ethanol for 5 min and once in distilled water for 5 min. After deparaffinization was completed, slides were stained using hematoxylin solution (indicated brand and city) dried in the air for approximately 10 min at room temperature. Before AFM analysis, each glass slide was cut to include the tumor area on a glass portion of 1 cm × 1 cm.

Before AFM imaging, the stained paraffin sections of the same tissue block were examined under the transmitted light illumination of the optical microscope (Primovert microscope) with a magnification of 40× to optically identify the M or NM tumor areas by inspection. Then, the AFM probe was positioned in relation to the section as identified by the optical image. The AFM imaging was performed in the air at ambient temperature.

### 2.2. AFM Image Analysis

The fixed histological tissues were imaged by Innova AFM (www.bruker.com) operating in tapping mode with phosphorus (n)-doped silicon cantilever (RTESPA, Bruker, Madison, 120 WI, USA) with a nominal tip diameter of 8–10 nm, and nominal spring constant of 40 N/m at 300 kHz resonance frequency. Surface image quality was optimized by lowering the scan rate at 0.2 Hz. All images were acquired with 50 × 50 μm^2^ scan sizes, 512 × 512 data point resolution, and with a pixel size of 97.65 nm. Each scanned sample contains a few CRCs since 11 μm is their median size [47]. The AFM was installed on a vibration isolation table (minus k technology BM-10) to compensate for regular environmental vibrations and placed inside an acoustic enclosure (Ambios technologies Isochamber) for isolation from thermal and building vibrations and 30 dB acoustic drift. The AFM imaging was performed in air at ambient temperature. In addition to height, the amplitude and phase images were also recorded.

### 2.3. AFM Image Mathematical Analysis

The AFM tip is moving along the x axis (scanning or fast axis). Each pixel has a surface of about 9600 nm^2^, which is much larger than the surface of the tip, and at each pixel the surface height–height roughness (z value) is recorded. The position of each pixel onto the surface is given by two indices *i* and *j*, where $i=1,2,3,\ldots ,N_{x}$ and $j=1,2,3,\ldots ,N_{y}$, with $N_{x}$ and $N_{y}$ are the maximum number of pixels in each direction. The available data for analysis are of the form f(x,y)=z or in discrete form $f\left(x_{i},y_{j}\right)=z_{ij}$. These data are first analyzed with MF-DFA [48,49] and then with GMM [50,51,52,53,54]. For the details of MF-DFA and GMM, see Appendix A and Appendix B respectively. Of note, any systematic error introduced by sample preparation, e.g., cut, does not affect either MF-DFA or GMM analysis; these are doing detrending, so any constant additive value is canceled out, as can also be seen in reference [54] and the supporting information therein.

## 3. Results

**Figure 1:**
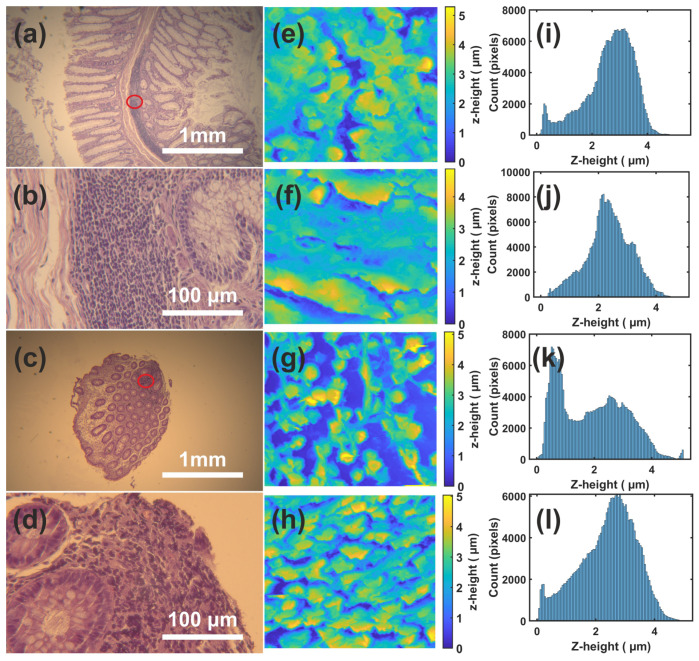
(**a**) Optical image of an M tissue in 4× lens; (**b**) the same as (**a**) for 40× magnification; (**c**) the same as (**a**) for NM tissue; (**d**) the same as (**b**) for the NM tissue; (**e**,**f**) AFM images of tiny parts (50 × 50 μm^2^) of the M tissue displayed in (**a**,**b**). (**g**,**h**) AFM images of tiny parts (50 × 50 μm^2^) of the NM tissue displayed in (**c**,**d**); (**i**) histogram of AFM tip elevation (z heights) illustrated in (**e**); and (**j**–**l**) the same as (**i**) for (**f**–**h**). Notice that, magnification 4× provides an analysis of 1920 × 1200 pixels (an area of 3.2 × 2 mm^2^), and 40× magnification provides the same analysis for an area of 320 × 200 μm^2^. Of note, the visual similarity of the AFM images (**e**,**f**), the M sample, which, however, lead to different histogram distributions.

Optical images (4×, 40×) of the CRC tumor histological cross-sections stained by hematoxylin/eosin reveal M differentiation, as shown in Figure 1a,b. Of note, they have a different morphology with respect to the NM tissues illustrated in Figure 1c,d. The size of the tumor surface is 0.17 × 1.73 mm^2^ already large and probably critical for patient survival, as shown in Figure 1a. A small portion, 50 × 50 μm^2^ of the tissues displayed in Figure 1b,d, were used for the AFM images, Figure 1e,h. Visual inspection underlines a large number of nano-islands for the NM compared to the M ones.

Additionally, a sequence of high-to-low height value (yellow-to-dark blue) is repeated all over the surface for NM sections, while the M ones follow a smoother distribution of heights. These criteria are subjective and cannot lead to discrimination between the two classes, as can be seen, in the similarity of the corresponding histograms shown in Figure 1i–l. Fifteen AFM images were analyzed; six of them correspond to NM and the rest to M cancerous samples. All samples are cross-sections of CRC tissues which were taken from five different patients, and more than one sample can belong to the same patient or/and to the same tissue.

AFM images, some of which are depicted in Figure 1e–h, were first analyzed by 2D-MFDFA, extracting the multifractal scaling exponent τ(q), and then reanalyzed by 2D-GMM, which provides the scaling exponent of the structure function, z(q). τ(q) and z(q), as can be seen in Equations (A5) and (A9) in Appendix A and Appendix B, respectively, contain all the necessary info for decoding multifractal processes. Through τ(q), ref. [55] two other metrics can be constructed: the singularity strength, α(q), and the multifractal spectrum, f(α), see Equation (A6) in Appendix A.

**Figure 2:**
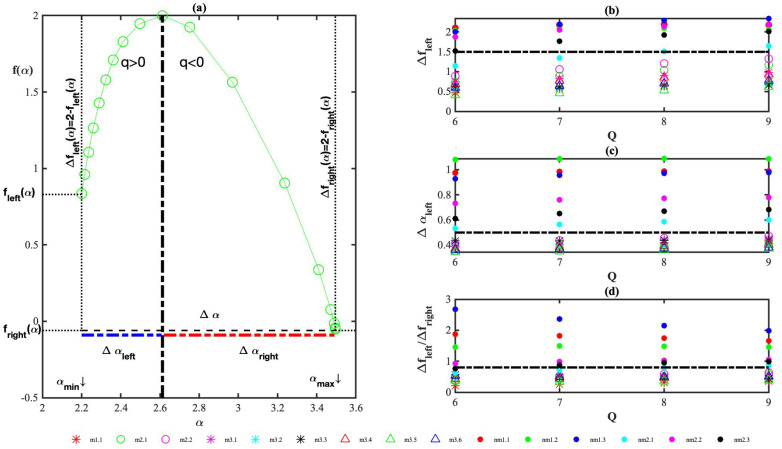
(**a**) Definition/visualization of the measures Δα, $\Delta \alpha_{i}$, $\Delta f_{i}$ (i=left/right) used to investigate multifractal spectrum asymmetry as well the singularity parameter asymmetry. The multifractal spectrum of the sample m2.1 was used for the definitions. The multifractal spectrum of NM samples is illustrated in Figure A1d; (**b**) width of multifractal spectrum to the left, $\Delta f_{left}$; (**c**) singularity parameter to the left, $\Delta \alpha_{left}$; and (**d**) ratio of singularity parameters to the left/right, $\Delta f_{left}/\Delta f_{right}$. In all graphs, Q stands for the maximum moment. Calculations were carried out for *q* in the range [−Q − 1, Q + 1]. The numbering of the samples is determined by an alphanumeric sequence whose first letter determines the assessment of the histopathologists by optical microscope, m/nm for metastatic/non-metastatic. The first number identifies the patient and the number after the dot the sample number, e.g., m1.2, means that the second sample of the first patient is metastatic.

**Figure A1:**
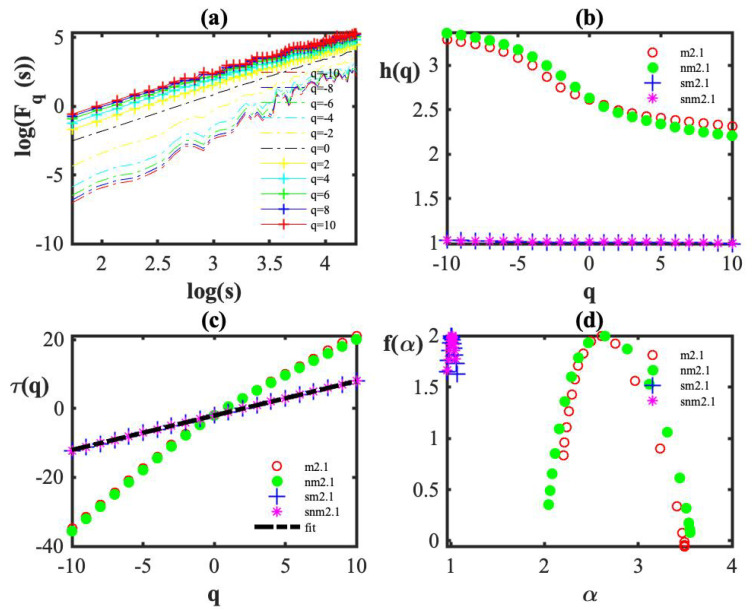
(**a**) $log(F_{q}\left(s_{x},s_{y}\right))$ versus log(s) for a randomly chosen sample, as can be seen in Equation (A4). (**b**) The scaling exponent, h(q), of $F_{q}\left(s_{x},s_{y}\right)$, Equation (A4), versus the order of the moment *q* is depicted. (**c**) The multifractal scaling exponent τ(q) as it is obtained via Equation (A5) versus the order of the moment, *q*, is illustrated. (**d**) The multifractal spectrum, f(α) versus α, as it is predicted via Equation (A6), is displayed. Figures (**b**–**d**), for clarity reasons, show the results for only two samples, namely m2.1 and nm2.1. The rest of the samples present similar behavior. At the same figures and for the same samples, the behavior of the shuffled data is depicted. The alphanumeric sequence of the shuffled data reads sm2.1 and snm2.1.

**Figure A2:**
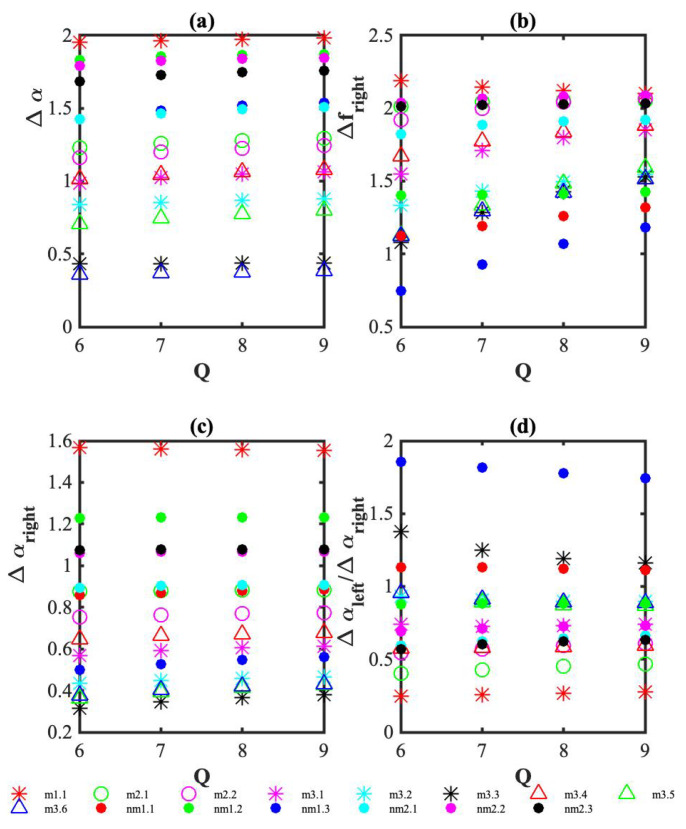
(**a**) $\Delta \alpha =\alpha_{max}-\alpha_{min}$ width of multifractal spectrum. Notice that, $\alpha_{max}=\alpha \left(-Q\right)$ and $\alpha_{min}=\alpha \left(Q\right)$; (**b**) $\Delta f_{right}$, right singularity parameter characterizing the broadness of the tail to the right of multifractal spectrum; (**c**) $\Delta \alpha_{right}=\alpha \left(q=0\right)-a\left(q\right)$, q<0, width of multifractal spectrum to the right; (**d**) $A=\Delta \alpha_{left}/\Delta \alpha_{right}$, informative descriptor of skewing the multifractal spectrum to the left or to the right. A>1 is a spectrum skewed to the right, and A<1 is a spectrum skewed to the right and symmetric for A=1.

**Figure A4:**
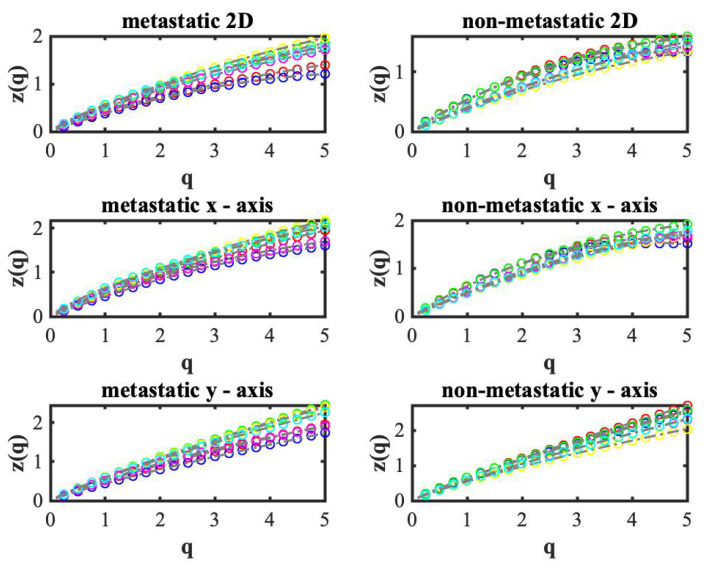
The scaling exponent of the structure functions for six M and six NM histological cross-sections, as was obtained by analyzing the surface roughness, as well as roughness anisotropy in the x and y axes.

**Figure A5:**
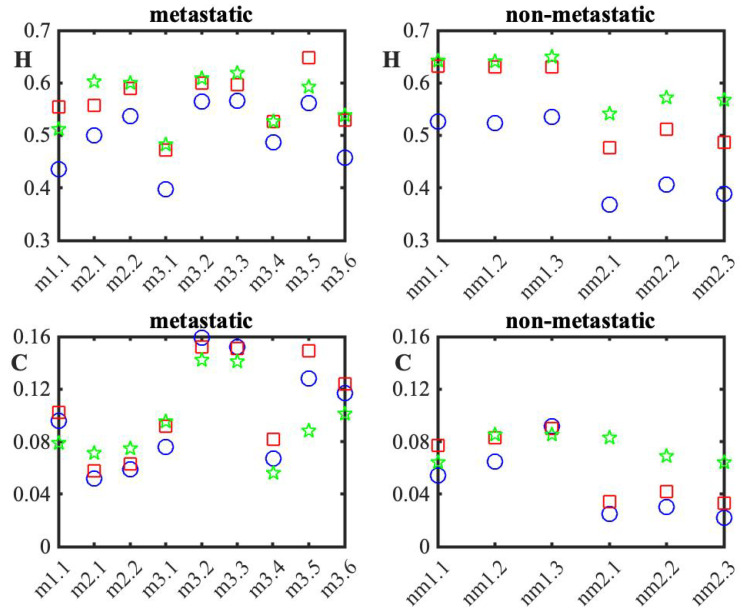
The obtained generalized Hurst exponent, *H*, and the co-dimension parameter, *C*, for two-dimensional analysis as well for analysis in separate x and y axes for M and NM histological cross-sections. Color code: blue for surface analysis; red for analysis in x axis; and green for analysis in the y axis.

The analysis by both MF-DFA and GMM in two dimensions showed that all AFM images (M and NM) pose a multifractal character whose origin emanates from the long-range correlations of weak and strong fluctuations, as can be seen in Figure A1 in Appendix C, where the behavior of the shuffling data is illustrated as well the discussion there. Furthermore, the histological cross-sections are not homogeneous, as revealed by GMM analysis in separate axes, and as can be seen in Figure A4 and Figure A5 and the discussion thereof in Appendix C. The obtained multifractal scaling exponents, τ(q), present minute differences for M and NM histological cross-sections. The multifractal spectrum or spectrum from now on, f(α), is mostly skewed to the right (q<0) than to the left (q>0), as can be seen in Figure 2a, whereas the width of the spectrum, Δα, is not enlightening in addressing the differences between M and NM histological sections, as can be seen in Figure A2a,d) and the discussion in Appendix C. Of note, Δα, has been successfully tested and was constituted the first response-predictive marker in stage II–III colon cancer [18]. The following measures were defined in quantifying the asymmetry properties of f(α). (i) $\Delta \alpha =\alpha_{max}-\alpha_{min}$ returns the width of the spectrum. (ii) $\Delta \alpha_{left}=\alpha_{max}-\alpha \left(q=0\right)$, where f(α(q=0))=2, returns the width of the spectrum to the left (q<0, weak fluctuations). (iii) $\Delta \alpha_{right}=\alpha \left(q=0\right)-\alpha_{min}$ the same as (ii) but to the right and accounts for strong fluctuations (q>0), and note $\Delta \alpha \left(q\right)=\Delta \alpha_{left}+\Delta \alpha_{right}$. (iv) $\Delta f_{left}=2-f_{left}$ returns the singularity parameter of the spectrum to the left. (v) $\Delta f_{right}=2-f_{right}$ the same as (iv) but to the right. Note that $f_{left}=f\left(\alpha_{max}\right)$ and $f_{right}=f\left(\alpha_{min}\right)$. All these measures are illustrated schematically in Figure 2a. In the same figure, the spectrum of a randomly chosen sample (m2.1) is depicted. A vertical dashed solid black line indicates the value of *a* for which f(α) takes its maximum value. This value, which is equal to 2, is the same for all samples and determines the fractal dimension of the support ($D_{f}$). Note that the maximum of f(α(q)) is achieved for q=0.

The singularity parameters $\Delta f_{i}$, i=left/right characterize the broadness of the spectrum and their ratio, $\Delta f_{left}/\Delta f_{right}$ is a direct measure of the depth of the spectrum tail. If $\Delta f_{left}/\Delta f_{right}>1$, the left-hand side is deeper (for q>0) and implies strong singularities, while for $\Delta f_{left}/\Delta f_{right}<1$, the tail for q<0 is deeper, implying weak singularities. If the spectrum was symmetric on both axes, then $\Delta \alpha_{left}=\Delta \alpha_{right}$ and $\Delta f_{left}=\Delta f_{right}$ would hold true. For $\Delta \alpha_{left}>\Delta \alpha_{right}$ and $\Delta f_{left}>\Delta f_{right}$, the spectrum is skewed to the left, and it is skewed to the right otherwise. Figure 2b–d show three measures that, in principle, can differentiate M from NM histological cross-sections. Every single point depicted in these figures corresponds to a full-2D-MF-DFA analysis for *q* in the range [−Q−1,Q+1], thus proving that the results have no dependence on the order of the moment *q*. In the present study, $Q_{min}=6$, and $Q_{max}=9$. Figure 2a shows the marker $\Delta f_{left}$(strong fluctuations) for M and NM histological sections, a horizontal black-dashed line shows the threshold value 1.5, for which $\Delta f_{left}<1.5$ for all M samples and the opposite for NM, thus indicating a stronger left singularity parameter for NM samples. Figure 2c shows that the marker $\Delta \alpha_{left}$. A dashed black line was set at the value of 0.5 with $\Delta \alpha_{left}<0.5$ for all M histological sections and the opposite for NM ones. The width of the spectrum to the left is constantly smaller for M sections and, by combining this with the behavior of the singularity parameter to the left, we draw our first argument which says that weak fluctuations likely govern M samples. Given that a proper measure should answer on the kind of cancer histological section without the possibility of comparison between M and NM ones, an index that compares contribution from weak and strong fluctuations and differentiates M from NM in a single run analysis is needed. $\Delta f_{left}/\Delta f_{right}$ is such a marker, as shown in Figure 2d, and it shows a clear differentiation among M and NM histological sections. The threshold value is 0.8 and all values higher than 0.8 indicate NM samples, and M otherwise. This marker is one of the main contributions of this work because of acting as a universal one in tackling the question of whether a histological colorectal cancer section is metastatic or not. We should notice that the measures $\Delta \alpha_{right}$, $\Delta f_{right}$ and $\Delta \alpha_{left}/\Delta \alpha_{right}$ are not conclusive. They are depicted in Figure A2b–d in Appendix C, where the relative discussion can also be seen.

Ensuring forecasting, an additional marker obtained by another method focusing on different properties, is required. If this measure exists, and a histological cross-section satisfies both markers simultaneously, then it will secure the success of the present concept for discriminating M from NM cancerous sections. 2D-GMM delivers the new desirable marker, which is actually identified as the multifractality index, $\alpha_{L}$. As mentioned above, 2D-GMM returns the scaling exponent of the structure function, z(q), whose form is then fitted by Equation (A10) of Appendix B, and the values of the generalized Hurst exponent, *H*, the co-dimension parameter, *C*, and the multifractality index are obtained. GMM was used for the analysis of surface raw data as well for raw data along each axis, and the obtained values are listed in Table A1 and z(q) is depicted in Figure A4, see Appendix C. The parameters *H* and *C* are not conclusive; see Figure A5 and the discussion in Appendix C.

**Figure 3:**
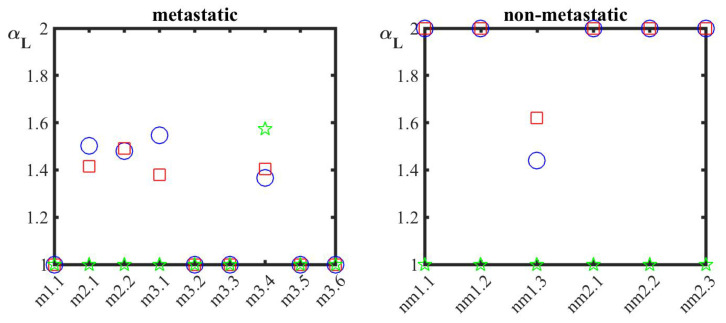
The multifractality index $\alpha_{L}$ obtained by the best fitting of z(q) by means of Equation (A10) or special cases of it, for the surface, as well for separate analysis in x and y axes. Symbols: blue circles for height–height surface roughness, red squares for surface roughness along x axis which is identified to the scanning one, and a green star for y axis.

A sound difference between M and NM samples is provided by the values of the multifractality index, $\alpha_{L}$, Figure 3. For NM sections, the value of $\alpha_{L}$ is equal to 2 (log-normal distribution), the exception being the sample nm1.3. The same is true for analysis along the x axis, where again, the exception of sample nm1.3 gives value 1.62, which is, however, larger than that for surface analysis. However, the sample nm1.3 satisfies the general trend for NM samples with respect to the parameters *H*, *C* and $a_{L}$, which define the structure function of a sample, as it holds true that $H_{y}\geq H_{x}\geq H_{surface}$, and $C_{y}\geq C_{x}\geq C_{surface}$, exception the nm1.1 sample. Of note, the value of $\alpha_{L}$ for analysis along the y axis is constantly equal to 1 (log-Cauchy distribution) without any exception, which it also holds true for M cells with the exception of sample 3.4. On the contrary, for M tissues, the values of $\alpha_{L}$ for surface or along x axis are either equal to 1 for 5 out of 9 samples or smaller than 1.5, with the surface multifractality index being higher than the corresponding index in the x axis. Thus, values of the multifractality index close or equal to 2 for analysis along the scanning axis (x axis) and 1 for analysis along the y axis underline the existence of a NM histological section. This is the second main contribution of the present work.

## 4. Discussion

MF-DFA and GMM showed that AFM tip response over a histological cross-section (height–height surface roughness) for both M and NM samples presents a multifractal character, whose origin emanates from long-range correlations. The sections are inhomogeneous because of the surface roughness which displays asymmetric scaling along the x and y axes. The multifractal spectrum is not symmetric but mostly skewed to the right. Based on spectrum asymmetry, a first discrimination between M and NM tissues may be made by comparing the width of the spectrum to the left and right, as well the singularity parameter to the left and right. The MF-DFA provides a universal marker, which quantifies contributions from weak and strong fluctuations and, according to its value, we can classify a histological cancer section as metastatic, or not. The width of the spectrum is mainly larger for NM sections, larger variability and thus more multifractal nature. NM prefers to be skewed to the right, however, the tail of their spectrum goes deeper to the left—implying stronger fluctuations, which in turn declare the existence of irregular areas and of some rare events of high values. On the contrary, M sections are right-skewed and the tail of the spectrum goes deeper to the right, implying weak fluctuations, which are associated with valleys (mostly flat areas).

Histological cross-sections are intrinsically built up by protrusions and valleys of different geometries and of different size orders. At cellular size level of some microns, this diverse geometry is revealed through its interaction with the AFM tip and returns for M tissues a roughly smooth section frequent distributed by valleys. The anaglyph remains the same for NM tissues; however, it is interrupted by some protrusions contributing to the high elevation of the AFM tip. Frequency histograms for NM cross-sections, as shown in Figure 1k,l, clearly illustrate the existence of some events, larger than 4 μm, contrary to what happens for the corresponding histograms for M cross-sections, Figure 1i,j. This finding is also compatible with the estimated value of the multifractality index, $\alpha_{L}=2$, for almost all samples whose higher values underline the existence of a small number of pixels, however, with stronger upward values.

The analysis also returned the value of $\alpha_{L}=1$ for all M and NM tissues, except one, along the y axis. Mathematically, $\alpha_{L}=1$ corresponds to z(q)=Hq−Cqlog(q) as the function describing the scaling exponent of the structure function, and underlines that fluctuations of surface roughness along the y axis can be considered as a collection of random variables whose logarithm follows Cauchy distribution, with fatter tails with respect to the Boltzmann one. Along the x axis, for almost all NM, $\alpha_{L}=2$ and accordingly $z\left(q\right)=Hq-C\left(q^{2}-q\right)$ indicate surface roughness fluctuations as a collection of random variables whose logarithm follows the log-normal distribution. Both log-Cauchy and log-normal distributions point to the multiplicative effects of different origins, and the latter reflects on the mechanisms that govern the growth of metastatic and non-metastatic cancerous tissues.

## 5. Conclusions

In summary, height–height surface roughness fluctuations of cancerous cross-sections of M and NM CRC tissues obtained by AFM measurements were analyzed by two powerful statistical approaches, namely MF-DFA and GMM. Analysis showed that cross-sections are multiaffine surfaces, and in general, the differences between M and NM are minute. However, the values of two cardinal metrics can serve as markers for distinguishing M from NM; (a) the ratio of the singularity parameters (left/right), which are defined relative to (weak/strong) fluctuations in the multifractal spectrum, is always greater than 0.8 for NM tissues; and (b) the index of multifractality describing the fluctuations across the scanning axis is equal to 2 for NM sections. This pair of values never occurs for M sections. The analysis of histological cancerous cross-sections can therefore safely identify the metastatic or non-metastatic nature of the tissue when both markers appear. These findings, and their understanding, will enhance our arsenal in the war against cancer.

## Figures and Tables

**Figure A3:**
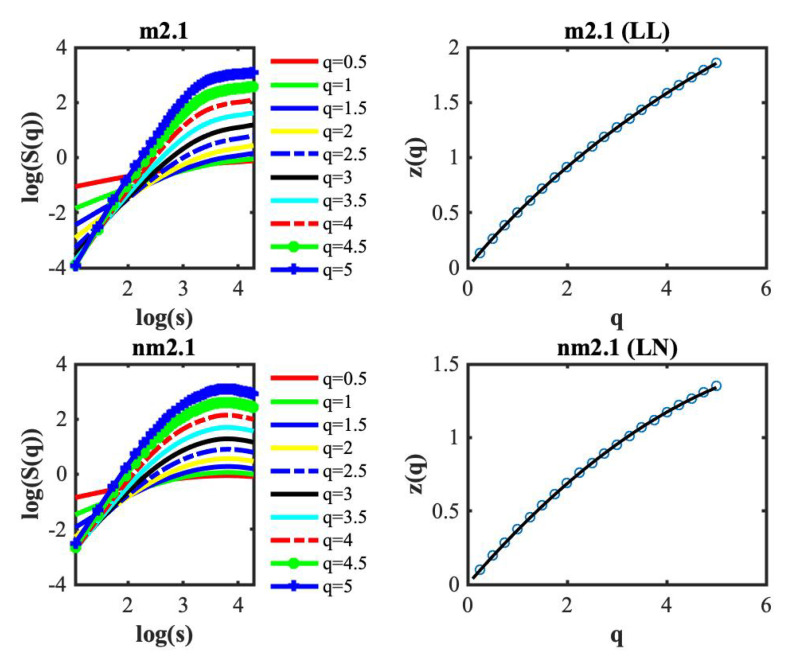
Linear regression of log(S(q)) versus log(s). For clarity reasons, the order of moments in the range [0.5,5] with a step of 0.5 are displayed. The actual calculations were carried out for moments in the range [0.25, 5] with a step of 0.25. The slope of each line corresponds to the value of the scaling exponent of the structure function, z(q). Two cases (m2.1 and nm2.1) are provided. The title (LL) for m2.1 means Log Levy, and (LN) for nm2.1 means Log Normal, as can be seen in the discussion below.

## Data Availability

The availability of height–height surface roughness data and software: all data that support the findings of this study are available from the corresponding author upon reasonable request, and in addition, the software (code) is available from the corresponding author in the flow chart upon reasonable request.

## Abbreviations

The following abbreviations are used in this manuscript:

FD	Fractal dimension
M	Metastatic cross-sections
NM	Non-metastatic cross-sections
2D MF-DFA	Two-dimensional multifractal detrended fluctuation analysis
2D GMM	Two-dimensional generalized moments method

## Appendix A. Multifractal Detrended Fluctuation Analysis in Two Dimensions (2D-MFDFA)

Let us assume that the data, $\left(x_{i},y_{j}\right)$, describe a self-affine surface. For the application of 2D-MFDFA, the following steps are taken, as in ref. [49]: (i) The surface is partitioned into $M_{s_{x}}$ × $M_{s_{y}}$ dis-joint surfaces, where $s_{x}=\frac{N_{x}}{M_{s_{x}}}$ and $s_{y}=\frac{N_{y}}{M_{s_{y}}}$ are the scales along the x and y axes respectively, and $N_{x}$, $N_{y}$ provide the maximum number of data along x and y axes. The coordinates of each sub-surface are given by $\left(x_{\left(k_{x}-1\right)s_{x}+i},y_{\left(k_{y}-1\right)s_{x}+j}\right)$, with $1\;\leq \;i\;\leq \;s_{x}$, $1\;\leq \;j\;\leq \;s_{y}$ and $k_{x}$ and $k_{y}$ are indices determining the position of each sub-surface and take values in the range, $k_{x}=1,2,\ldots ,M_{s_{x}}$ and $k_{y}=1,2,\ldots ,M_{s_{y}}$. If $N_{x}=N_{y}$, the square surface, then $s_{x}=s_{y}=s$ is the scale, and $M_{sx}=M_{sy}=M_{s}$ provides the number of dis-joint squares after the partitioning of the whole surface by the scale *s*, and the coordinates of each sub-square are $\left(x_{(k_{x}-1)s+i},y_{(k_{y}-1)s+j}\right)$. Given that data correspond to square surfaces, in the rest of the paper, we keep the notations for square surfaces; and (ii) for each sub-square, we define the cumulative sum as follows

(A1) $$u_{k_{x},k_{y}}\left(x_{n},y_{m}\right)=\sum_{i=1}^{n}\sum_{j=1}^{m}\left(x_{\left(k_{x}-1\right)s_{x}+i},y_{\left(k_{y}-1\right)s_{y}+j}\right)$$

with 1 ≤ n,m ≤ s. $u_{k_{x},k_{y}}\left(x_{n},y_{m}\right)$ is again a surface, whose trend can be removed by a bivariate polynomial. (iii) detrending step: Various forms of functions for detrending a surface have been proposed [49]. We use a simple plane, ${\bar{u}}_{k_{x},k_{y}}\left(x_{n},y_{m}\right)=ax+by+c$. The parameters a,b,c can be easily obtained by linear regression. We define the residual matrix as $\epsilon_{k_{x},k_{y}}\left(x_{n},y_{m}\right)=u_{k_{x},k_{y}}\left(x_{n},y_{m}\right)-{\bar{u}}_{k_{x},k_{y}}\left(x_{n},y_{m}\right)$. The detrended fluctuation function of the sub-square is provided as the variance of the residual matrix [56].

(A2) $$F\left(k_{x},k_{y},s_{x},s_{y}\right)=\sqrt{\frac{1}{s^{2}}\sum_{i=1}^{s}\sum_{j=1}^{s}\epsilon_{k_{x},k_{y}}^{2}\left(x_{\left(k_{x}-1\right)s_{x}+i},y_{\left(k_{y}-1\right)s_{y}+j}\right)}$$

(iv) the last step considers the estimate of the $q_{th}$ order of the detrended fluctuation function, which reads

(A3) $$F_{q}\left(s_{x},s_{y}\right)=\left\{\begin{array}{c} {\left(\frac{1}{M_{x}M_{y}}\sum_{k_{x}=1}^{M_{x}}\sum_{k_{y}=1}^{M_{y}}F^{q}\left(k_{x},k_{y},s_{x},s_{y}\right)\right)}^{\frac{1}{q}},q\neq 0\\ \frac{1}{M_{x}M_{y}}\sum_{k_{x}=1}^{M_{x}}\sum_{k_{y}=1}^{M_{y}}ln\left(F\left(k_{x},k_{y},s_{x},s_{y}\right)\right),q=0 \end{array}\right.$$

The power law relation between $F_{q}\left(s_{x},s_{y}\right)$ and the scale ${\left(s_{x}^{2}+s_{y}^{2}\right)}^{\frac{1}{2}}$, reads

(A4) $$F_{q}\left(s_{x},s_{y}\right)∼{\sqrt{s_{x}^{2}+s_{y}^{2}}}^{h(q)}\overset{s_{x}=s_{y}=s}{∼}{\left(\sqrt{2}s\right)}^{h(q)}$$

The multifractal scaling exponent or mass function, τ(q), is the key function in 2D-MFDFA, and reads

(A5) $$\tau \left(q\right)=qh\left(q\right)-D_{f}$$

where $D_{f}$ is the fractal dimension of the substrate support, equal to 2 for surfaces, h(q) is given by Equation (A4), and *q* stands for the order of the moment taking values in the range [−10,10]. Negative values of *q* ascribe the contribution of weak fluctuation and positive values those of strong fluctuations. If τ(q) is a linear function of *q*, the process is monofractal. Convex shape of τ(q) versus *q* signals to a multifractal process. Linearity by portions, e.g., a bilinear form of τ(q) versus *q* does not necessarily point to a multifractal process [57]. The singularity strength, α(q), and the multifractal spectrum, f(α), are connected to τ(q) via the Legendre transform [55]

(A6) $$\begin{array}{c} \alpha \left(q\right)=\frac{d\tau (q)}{dq}\\ f(\alpha )=q\alpha -\tau (q) \end{array}$$

For monofractal processes, the multifractal spectrum is just a single point. Instead, multifractal processes are characterized by a concave spectrum and the broader the shape of the curve, the more multifractal the process is. Additionally, the spectrum being skewed to the left or to the right and/or differences in the broadness of the spectrum to the left with respect to the right or vice versa can differentiate complex processes.

## Appendix B. Generalized Moments Method in Two Dimensions (2D-GMM)

Fluctuation analysis in two dimensions, with the generalized moments method (2D-GMM), can be seen as an extension of the height–height correlation function [58] because it takes into account various moments also including fractional ones. The method has accurately described the kinematics of molecules on surfaces [51], it has shed light on cells’ behavior in complex environments [50,52,53,59] and it has unveiled the biomemory effects caused by AFM tip penetration into spore cells’ surface [54]. The 2D-GMM is used to classify the multiaffine properties of surface height–height roughness, and takes as input a finite set of spatial data of typically regular locations (2D-lattice). $z_{i,j}$ is the surface roughness with i,j being the x and y coordinates of the corresponding pixel, respectively. Fluctuation analysis considers the absolute value of the difference of the surface roughness between pixels spaced apart by $\sqrt{s_{x}^{2}+s_{y}^{2}}$.

(A7) $$\mu \left(s_{x},s_{y}\right)={\left({\left(z_{s_{x}+i,s_{y}+j}-z_{i,j}\right)}^{2}\right)}^{1/2}$$

with $i=1,2,\ldots ,N_{x}-s_{x}$, $j=1,2,\ldots ,N_{y}-s_{y}$, and for $s_{x}=2,3,\ldots ,N_{x}/10$, $s_{y}=2,3,\ldots ,N_{y}/10$ in order for the ensemble average to be statistical reliable. Note that $N_{x}$, $N_{y}$ provide the maximum number of data along the x and y axes. Equation (A7) can be seen as an unweighted probability measure of surface roughness. We define the structure function as the ensemble average of $\mu {\left(s_{x},s_{y}\right)}^{q}$, which for discrete datasets, reads as time average

(A8) $$S_{q}\left({\left(s_{x}^{2}+s_{y}^{2}\right)}^{1/2}\right)=\frac{\sum_{i=1}^{N_{x}-s_{x}}\sum_{i=1}^{N_{y}-s_{y}}{\left(\mu \left(s_{x},s_{y}\right)\right)}^{q}}{\left(N_{x}-s_{x}\right)\left(N_{y}-s_{y}\right)}$$

The structure function defined in Equation (A8) follows a power-law dependence

(A9) $$S_{q}\left({\left(s_{x}^{2}+s_{y}^{2}\right)}^{1/2}\right)∼{\left({\left(s_{x}^{2}+s_{y}^{2}\right)}^{1/2}\right)}^{z(q)}\overset{s_{x}=s_{y}=s}{=}{\left(\sqrt{2}s\right)}^{z(q)}$$

where z(q) is the scaling exponent and its shape dependence on *q* classifies a process either as monofractal (linear) or as multifractal (convex shape). The small order moments, 0<q<2, are responsible for the core of the probability density function (pdf) and higher moments contribute to tails of a pdf [60], and notice that q>0 in GMM. Among multifractals, universal multifractals are ubiquitous [61],

(A10) $$z\left(q\right)=qH-\frac{C}{\alpha_{L}-1}\left(q^{\alpha_{L}}-q\right)$$

The scaling exponent contains a linear term in *q* (monofractal) and a multifractal correction to it (non-linear term), which leads the departure from linearity. For the linear dependence of z(q) on *q*, H=z(q=1) is called the Hurst exponent in honor of Edwin Hurst [62], and in general, for multifractals, it is called the generalized Hurst or Hölder exponent. For z(q)=Hq, the fluctuations describe a quasi-Gaussian field. For a log-Levy model, as described by Equation (A10), 0<H<1, the value of *H* shows the degree of fractional integration (persistent for H>0.5, antipersistent for H<0.5). H=0 describes a conservative filed, scale invariant. The term *C* is defined as $C=\frac{dz(q)}{dq}{|}_{q=1}-H$, is called co-dimension information and measures the mean intermittency; for C=0, the field is homogeneous, and only one scale exists. From a lower *C* to higher *C*, the degree of intermittency increases, and some extreme outliers will occur. $\alpha_{L}=-\frac{1}{C}\frac{d^{2}z\left(q\right)}{dq^{2}}{|}_{q=1}$ is the Levy index or index of multifractality; it takes values in the range $0\leq \alpha_{L}\leq 2$, and for $\alpha_{L}=0$, returns Normal distribution. For $\alpha_{L}=2$, $z\left(q\right)=Hq-C\left(q^{2}-q\right)$ and the logarithm of the field is normally distributed [63]. For $\alpha_{L}=1$, z(q)=Hq−Cqlog(q) and the field is distributed according to log-Cauchy. For all the other values of $\alpha_{L}$ in the range [0,2], the field is distributed according to log-Levy. Notice that Equation (A10) describes a monofractal field, that is, a homogeneous field with a unique scaling, only if C=0 or $\alpha_{L}=0$. The multifractality index $\alpha_{L}$ is used to characterize the multifractal intensity, which reflects the hierarchical structure of a process. For a surface roughness field, lower values of $\alpha_{L}$ reflect a surface having more pixels with high values, and higher values of $\alpha_{L}$ mirror fewer pixels but with stronger upward values.

Of note, the relation between z(q), Equation (A10), and h(q), Equation (A4), respectively, is as follows:

(A11) $$h\left(q\right)=D_{f}+z\left(q\right)/q,q>0$$

## Appendix C. Analysis of Raw Data

MF-DFA and GMM investigate the scaling properties in a window of size $s_{x},s_{y}$, whose dimensions increase up to a certain length, not larger than N/10 with *N* being the maximum number of data along a given direction, in order to have statistical reliable results [48]. In the Cartesian coordinates used to define AFM tip motion, the x axis is the scanning one, the second one of the surface is the y axis, and the response of the tip is made along the z axis. Furthermore, we restrict the shape of the scaling window to be always a square one, $s_{x}=s_{y}=s$, where 4≤s≤N/10 with N=512. The side of the minimum square lag or scale used here is equal to 390.6 nm (4 ×97.65), where 97.65 nm is the size of the pixel, and $s_{x}=s_{y}=4$, see Equation (A4). The multifractal scaling exponent, τ(q), and scaling exponent of the structure function, z(q), Equations (A5) and (A10), respectively, contain all the necessary info for decoding multifractal processes. Through τ(q) [55], two other useful metrics can be constructed: the singularity strength, α(q), and the multifractal spectrum, f(α), as can be seen in Equation (A6). All measures are functions of the order of the moment, *q*, which for multifractal detrended fluctuation analysis, takes values in the range of [−Q−1,Q+1] with $Q_{min}=6$ and $Q_{max}=9$ in the current analysis. For GMM (fluctuation analysis), q∈(0,5] also takes non-integer values.

Figure A1a shows for each moment *q*, q∈[−10,10] the slope of $log(F_{q}\left(s_{x},s_{y}\right))$ versus log(s), which determines the value of h(q), as shown in (Equation (A4)). h(q) follows a decreasing trend as we pass from negative to positive moments, as shown in Figure A1b. The non-constant dependence of h(q) on *q* is a sign of multifractality. By making use of Equation (A5), we estimate the multifractal scaling exponent, τ(q), for the whole range of the moments used in the analysis. Figure A1c shows the form of τ(q) for m2.1 and nm2.1 histological cross-sections, and the rest of the samples follow similar behavior and figures are not shown. Its form either for M or for NM has a convex shape and both are pretty close to each another. The departure from linearity (monofractal surface) confirms multifractality, as the latter was already indicated by the form of h(q), as shown in Figure A1b. The multifractal spectrum, f(α) versus α, with the use of Equation (A6), as shown in Figure A1d, substantially differs from a single point, which is the identity of a monofractal surface. Additionally, the multifractal spectrum is skewed to the left or to the right and mainly shows a preference of curves skewed to the right (q<0) for both M and NM histological cross-sections. The broad probability distribution of the values of the discrete datasets and/or the long-range correlations of small and large fluctuations are the causes behind the onset of multifractality [48].

The trick of shuffled sequences is used to discriminate the origin of multifractality. We created shuffled sequences of the recorded height–height surface roughness data by randomly redistributing the elements of the original sequences and thus destroying all correlations. If for the shuffled data, $h_{shuf}\left(q\right)=const$ (const=1, for surfaces), then long-range correlations are responsible for the multifractal character. On the other hand, if $h_{shuf}\left(q\right)∼h\left(q\right)$, then multifractality originates from broad probability distribution because the latter is not affected by random redistributions. If both types of multifractality are present, then the shuffled data exhibit a weaker multifractality than the original one. The value of h(q=2) gives information about the degree of correlation. For uncorrelated or short-range correlated data, h(q=2)∼1.0. For h(q=2)<1.0, the data are anti-correlated and for h(q=2)>1.0, data are correlated and this correlation becomes stronger the higher the value of h(q=2) is. Of note, uncorrelated data are characterized by $h\left(q=2\right)=0.5D_{f}$ [53]. For all histological cross-sections, M and NM, h(q=2)>2 thus indicating a strong correlation. The values of this measure cannot discriminate M from the NM ones, as can be seen below in the discussion of the scaling exponent of the structure function z(q) whose values are listed in Table A1. In Figure A1b, the values of $h_{shuf}\left(q\right)$ for two samples are shown, and for all samples, this value is equal to 1. The multifractal scaling exponent for shuffling data, $\tau_{shuf}\left(q\right)$, is a linear function of *q*, and is depicted in Figure A1c, where the best fit on it has also been drawn—a black dashed line—and it has the form $\tau_{shuf}\left(q\right)=-k+q$, *k* is a constant and equal to $D_{f}$, as can be seen in Equation (A5). The multifractal spectrum of m2.1 and nm2.1 samples is shown in Figure A1d, where the corresponding spectrum of the shuffled data is also displayed, that is, small peaks close to the value of α=1. The rest of the samples follow the same behavior and are not shown. Summarizing the behavior of shuffling data; for all histological cross-sections, we have $h_{shuf}\left(q\right)=1$, $\tau_{shuf}\left(q\right)=-2+q$, and $f_{shuf}\left(\alpha \right)∼2$, which confirm the lack of multifractality for the shuffling data, and point to long-ranged correlations as a source of multifractality.

Figure A2 shows all measures defined to quantify the asymmetry and the broadness of the multifractal spectrum and is not conclusive in distinguishing M from NM histological cross-sections. In Figure A2a, the width of the multifractal strength, Δα, is presented. The higher the value of Δα, the more multifracatal the cross-section is. For NM sections, Δα>1.5, while the opposite is true for M sections. However, it cannot be used as a classification marker because of the exception of m1.1, which presents the higher value for this index among all samples. Given that Δα successfully predicted the prognosis and treatment response in stage II-III CRC [18], its ability to classify histological cross-sections according to their metastatic or non-metastatic nature, will be re-evaluated in future works. Note that every single point presented in these figures corresponds to MF-DFA analysis for *q* in the range [−Q−1,Q+1] and the associated measures do not have dependency on the order of the moment. Figure A2b presents the right singularity parameter. There is an overlapping of the results for M and NM histological cross-sections and by itself, it cannot stand as a discrimination index. The width of the multifractal spectrum for positive moments is illustrated in Figure A2c, where again it is a marker that cannot differentiate M from NM sections. The asymmetry parameter, A=$\Delta \alpha_{left}/\Delta \alpha_{right}$, is presented in Figure A2d. All M samples are skewed to the right, with the exception of the m3.3 histological cross-section. The majority of NM samples are also skewed to the right, exceptions being nm1.1 and nm1.3 which belong to the same histological section of the same patient. Of note, nm1.2 also belongs to the same histological cross-section and is coming from the same patient but shows an almost symmetric spectrum with a small tendency to the right. All measures presented in Figure A2 indicate the rather high similarity between M and NM histological sections, which can serve as descriptors for some properties of the multifractal spectrum but cannot serve as discrimination markers.

**Table A1:** The generalized Hurst exponent *H*, the co-dimension information *C* and the multifractality index $\alpha_{L}$ of the structure function scaling exponent, z(q). Note that $\alpha_{L}$ is not associated with an error when the best fit either obtained by $z\left(q\right)=Hq-C\left(q^{2}-q\right)$ log-normal for $\alpha_{L}=2$, or by z(q)=Hq−Cqlog(q) log-Cauchy for $\alpha_{L}=2$.

	2D-GMM			GMM (x-Axis)			GMM (y-Axis)		
**Sample**	**H**	**C**	$\alpha_{L}$	**H**	**C**	$\alpha_{L}$	**H**	**C**	$\alpha_{L}$
m1.1	0.435±0.003	0.096±0.002	1	0.554±0.002	0.102±0.002	1	0.512±0.003	0.079±0.002	1
m2.1	0.500±0.0003	0.052±0.0005	1.502±0.008	0.557±0.0006	0.058±0.0009	1.416±0.014	0.602±0.002	0.071±0.001	1
m2.2	0.536±0.003	0.059±0.0007	1.481±0.009	0.589±0.001	0.063±0.001	1.492±0.021	0.599±0.002	0.075±0.002	1
m3.1	0.397±0.002	0.076±0.003	1.546±0.038	0.472±0.004	0.092±0.005	1.379±0.055	0.482±0.003	0.095±0.002	1
m3.2	0.565±0.006	0.159±0.004	1	0.599±0.005	0.152±0.002	1	0.609±0.004	0.142±0.003	1
m3.3	0.566±0.006	0.152±0.004	1	0.596±0.004	0.151±0.003	1	0.619±0.005	0.141±0.004	1
m3.4	0.489±0.001	0.067±0.002	1.366±0.030	0.526±0.002	0.082±0.003	1.404±0.035	0.526±0.001	0.056±0.002	1.573±0.035
m3.5	0.562±0.004	0.128±0.003	1	0.648±0.004	0.149±0.003	1	0.593±0.002	0.088±0.002	1
m3.6	0.457±0.004	0.117±0.003	1	0.529±0.005	0.124±0.003	1	0.538±0.003	0.101±0.002	1
nm1.1	0.526±0.001	0.054±0.0003	2	0.632±0.001	0.077±0.0005	2	0.642±0.003	0.064±0.002	1
nm1.2	0.523±0.003	0.065±0.001	2	0.631±0.002	0.083±0.0008	2	0.640±0.004	0.085±0.003	1
nm1.3	0.535±0.0007	0.092±0.001	1.440±0.010	0.631±0.0003	0.090±0.0004	1.620±0.004	0.649±0.004	0.085±0.003	1
nm2.1	0.368±0.0008	0.025±0.0003	2	0.476±0.0006	0.034±0.0002	2	0.541±0.001	0.083±0.001	1
nm2.2	0.406±0.001	0.030±0.0004	2	0.511±0.002	0.042±0.0006	2	0.572±0.0008	0.069±0.0006	1
nm2.3	0.389±0.0004	0.022±0.0001	2	0.487±0.0004	0.033±0.0001	2	0.567±0.001	0.064±0.0008	1

We further investigated the role of multifractality by using 2D GMM in analyzing the raw data. Figure A3 shows the dependence of the structure function S(q) on the scale, *s*, 2≤s≤N/10, in log–log scale. The scale *s* is converted to μm by being multiplied by the side of the pixel, 97.65 nm. The two samples are illustrated, that is, m2.1 (upper panel) and nm2.1 (bottom panel). For different $q^{\prime }s$, the slope provides the value of the scaling exponent of the structure function, z(q), which is depicted in the graph on the right for each one of the samples presented here. z(q) departures from linearity and its convex shape underlines a multifractal surface in line with the findings of 2D MF-DFA.

Independently of the type of neoplasm and independent if we analyze the surface roughness or roughness across a specific direction, all corresponding to z(q), 12 of which are illustrated in Figure A4 have convex shape, which confirms their multifractal character. For each sample, we obtained the scaling of height–height roughness for the surface as well as for the x and y axes investigating the isotropy/anisotropy of the surface in parallel. The scaling exponent, z(q), was fitted with Equation (A10) (Log-Levy, general case), as well with equations $Hq-C(q^{2}-q)$ (Log-Normal) and Hq−Cqlog(q) (Log-Cauchy), which are specific cases of Equation (A10) for $\alpha_{L}=2$ and $\alpha_{L}=1$, respectively. The best fits are also presented in Figure A4, gray-dashed lines, and their values are listed in Table A1. Surface anisotropy because of the different z(q) along the x and y axes is even visible at the naked eye for NM histological cross-sections, while for M cross-sections, we conclude the same in the light of the analytical form of z(q), Figure A4.

The values of *H* and *C* are shown in Figure A5. For the surface, the generalized Hurst exponent is constantly smaller than the corresponding values for histological cross-sections’ roughness in the x and y axes. Furthermore, for NM cross-sections, the value of *H* along the y axis is always greater or equal than the corresponding value in the x axis. The same is true for most of the M samples, exceptions being samples m1.1 and m3.5. The co-dimension information, *C*, presents a differentiation between M and NM cells. For NM cells, *C* is constantly smaller for the surface than the corresponding values for scaling in x and y axes. On the other hand, for M cells, the values of *C* for both surface analysis and for individual analysis on the x and y axes do not show any systematic trend.
